# Ovarian Mechanobiology: Understanding the Interplay Between Mechanics and Follicular Development

**DOI:** 10.3390/cells14050355

**Published:** 2025-02-28

**Authors:** Haiyang Wang, Liuqing Yang

**Affiliations:** 1 Mechanobiology Institute, National University of Singapore, 5A Engineering Drive 1, Singapore 117411, Singapore; 2 NUS Bia-Echo Asia Centre of Reproductive Longevity and Equality, Yong Loo Lin School of Medicine, National University of Singapore, Singapore 117456, Singapore

**Keywords:** ovarian mechanobiology, follicular development, extracellular matrix, mechanotransduction

## Abstract

The ovary is a dynamic organ where mechanical forces profoundly regulate follicular development, oocyte maturation, and overall reproductive function. These forces, originating from the extracellular matrix (ECM), granulosa and theca cells, and ovarian stroma, influence cellular behavior through mechanotransduction, translating mechanical stimuli into biochemical responses. This review explores the intricate interplay between mechanical cues and ovarian biology, focusing on key mechanosensitive pathways such as Hippo signaling, the PI3K/AKT pathway, and cytoskeletal remodeling, which govern follicular dormancy, activation, and growth. Additionally, it examines how ovarian aging disrupts the mechanical microenvironment, with ECM stiffening and altered mechanotransduction contributing to a decline in ovarian reserve and reproductive potential. Emerging technologies, including 3D culture systems and organ-on-chip platforms, are highlighted for their ability to replicate the ovarian microenvironment and advance drug discovery and therapeutic interventions. By integrating mechanobiological principles, this review aims to enhance our understanding of ovarian function and provide new strategies for preserving fertility and combating infertility.

## 1. Introduction

The ovary is central to female reproductive health, orchestrating the production of oocytes and the regulation of hormonal balance throughout a woman’s reproductive lifespan. At the heart of ovarian function lies folliculogenesis, a dynamic process in which dormant primordial follicles sequentially activate, grow, and mature into ovulatory follicles capable of producing fertilizable oocytes [1,2]. This intricate process is tightly regulated by both intrinsic factors, such as the health of oocytes and granulosa cells (GCs), and extrinsic influences, including the extracellular matrix (ECM) and the ovarian stromal microenvironment [3,4]. However, as women age, ovarian function deteriorates due to structural and functional changes that compromise follicular development. Ovarian aging, marked by a decline in follicle quantity and quality, underlies natural reproductive decline and poses a significant challenge for women delaying childbirth [4,5]. This phenomenon manifests as diminished ovarian reserve, reduced oocyte competence, and infertility.

Recent advancements in mechanobiology—the study of how mechanical forces influence cellular behavior—have revealed that the ECM is not merely a structural scaffold but an active regulator of ovarian physiology [6,7,8]. The ECM transmits mechanical cues that govern key processes such as follicular dormancy, activation, and growth, largely mediated by mechanosensitive pathways like Hippo and PI3K/AKT. These pathways translate mechanical signals into biochemical responses, ultimately shaping follicular fate [9,10]. However, age-related ECM remodeling, characterized by increased fibrosis, excessive collagen deposition, and reduced elastin content, leads to heightened tissue stiffness [8,11,12]. This altered mechanical environment disrupts mechanotransduction pathways, impairing the balance between dormancy and activation. Such dysregulation accelerates follicular atresia, weakens oocyte–GC communication, and undermines overall ovarian function [11,13]. The intricate interplay between the ECM structure and cellular mechanotransduction forms a critical link between tissue mechanics and reproductive aging. By unraveling these mechanobiological mechanisms, researchers can identify novel targets for therapeutic interventions aimed at preserving ovarian function and improving reproductive outcomes in aging women.

This review explores the pivotal role of mechanobiology in ovarian function, with a focus on its influence on primordial follicle dormancy, activation, and antral follicle development. It delves into the impact of aging on ECM composition and mechanics, highlighting how these changes disrupt follicular dynamics and ovarian reserve. This review further examines emerging therapeutic strategies, such as in vitro activation (IVA) and whole ovary laparoscopic incision (WOLI), alongside advanced 3D culture systems, which offer novel avenues to counteract ovarian aging. By synthesizing insights from cellular biology, tissue mechanics, and reproductive medicine, this review aims to advance our understanding of ovarian mechanobiology and pave the way for innovative fertility-preserving therapies.

To ensure a comprehensive and up-to-date analysis, we conducted a systematic literature search using the PubMed database, covering studies published up to February 2025. The search utilized keywords such as ovarian mechanobiology, extracellular matrix in follicular development, mechanotransduction in oocytes, Hippo signaling in ovarian function, the PI3K/AKT pathway in folliculogenesis, ovarian aging and ECM stiffness, and mechanopharmacology in reproductive medicine. Relevant studies were selected based on their contribution to the mechanobiological understanding of folliculogenesis, with a focus on in vitro, ex vivo, and in vivo models.

## 2. Mechanobiology in Primordial Follicle Dormancy and Activation

**Figure 1:**
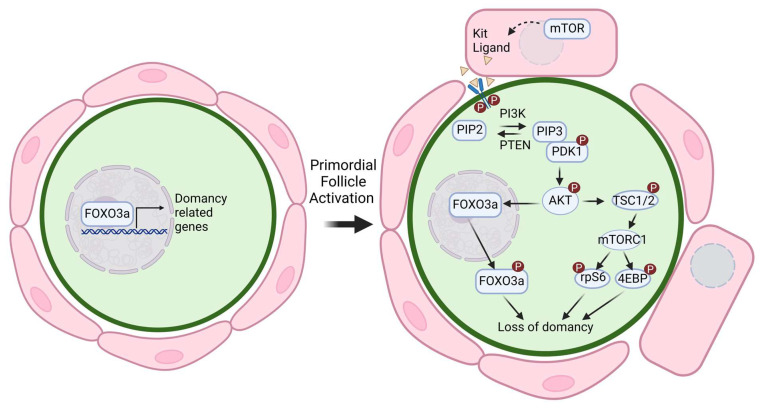
The PI3K/AKT signaling pathway is a key regulator of primordial follicle activation. Granulosa cell-induced activation of the mammalian target of the rapamycin (mTOR) pathway stimulates the secretion of Kit ligand, which binds to its c-KIT receptor on oocytes, initiating the phosphoinositide 3-kinase (PI3K) cascade. PI3K catalyzes the conversion of phosphatidylinositol-4,5-bisphosphate (PIP2) into phosphatidylinositol-3,4,5-triphosphate (PIP3), recruiting phosphoinositide-dependent kinase 1 (PDK1) to phosphorylate and activate AKT. Activated AKT translocates to the nucleus, where it phosphorylates and inhibits the transcription factor FOXO3, leading to its nuclear export and suppression of its dormancy-maintaining functions, thereby promoting primordial follicle activation. AKT also phosphorylates tuberous sclerosis complex 1/2 (TSC1/2), further activating mTOR complex 1 (mTORC1). mTORC1, in turn, regulates downstream effectors such as S6 kinase (S6K), ribosomal protein S6 (rpS6), and eukaryotic translation initiation factor 4E-binding protein (4EBP), promoting protein synthesis, granulosa cell proliferation, and overall follicular development. Created with BioRender.com.

The establishment and maintenance of primordial follicle dormancy are crucial for preserving the ovarian reserve and ensuring reproductive longevity in females. Primordial follicles, formed during embryogenesis, arise when female primordial germ cells aggregate into cysts. Postnatally, these cysts break apart as pre-granulosa cells and infiltrate and encapsulate individual germ cells, establishing dormant primordial follicles [14,15]. This dormancy is a highly regulated process, reliant on intricate communication between oocytes, GCs, and the extracellular environment (Figure 1). Understanding the mechanobiological mechanisms governing follicular dormancy and activation is vital for identifying strategies to optimize ovarian reserve and enhance reproductive outcomes.

### 2.1. ECM and Mechanical Forces in Dormancy Maintenance

Primordial follicles are primarily located in the ovarian cortex, a region enriched with ECM components that provide essential structural integrity, mechanical support, and biochemical regulation [16,17]. The composition of the ECM varies across follicular stages, influencing folliculogenesis through distinct molecular interactions. In the ovarian cortex, key ECM components include type I and type III collagen, which maintain tissue stiffness; type IV collagen and laminins, which form the basement membrane surrounding follicles; fibronectin, which mediates cell adhesion; and proteoglycans, such as decorin and perlecan, which modulate growth factor signaling. These ECM components play pivotal roles in follicular dormancy, activation, and maintenance.

The ECM serves as a mechanical niche, generating compressive forces that are critical for maintaining the quiescent state of primordial follicles. These forces regulate intracellular signaling pathways within oocytes, including those mediated by the transcription factor FOXO3, a central regulator of follicular dormancy [18,19]. In *Foxo3* knockout mice, primordial follicles are assembled normally but undergo rapid, global activation, leading to premature follicular depletion, ovarian failure, and infertility [18]. This underscores the indispensable role of FOXO3 nuclear localization in preserving follicular dormancy.

Mechanical forces derived from the ECM directly influence FOXO3 localization and activity. The disruption of ECM integrity, such as collagen degradation, leads to the cytoplasmic translocation of FOXO3, triggering follicular activation. Conversely, external mechanical pressure has been shown to restore FOXO3 nuclear localization, thereby preserving follicular dormancy [17]. These findings underscore the role of ECM-derived compressive stress in stabilizing the dormant state of primordial follicles by linking physical cues to intracellular regulatory mechanisms. Mechanistically, the ECM induces the formation of contractile stress fibers in squamous GCs, which generate compressive forces on the enclosed oocytes [17]. Remarkably, the nuclei of oocytes in primordial follicles respond to these mechanical forces by rotating. Interrupting this nuclear rotation leads to FOXO3 export to the cytoplasm, initiating oocyte activation and follicular growth.

Recent findings further reveal a spatial element in ECM-mediated dormancy maintenance [20]. Primordial follicles closest to ovulatory follicles are more likely to be activated due to localized ECM degradation and remodeling during ovulation. Specifically, the enzyme Cathepsin L (CTSL) plays a key role in degrading ECM components around ovulatory follicles, reducing mechanical stress on nearby primordial follicles and triggering their activation in a distance-dependent manner [20]. These findings demonstrate how ECM remodeling during physiological processes like ovulation impacts follicular dormancy and activation dynamics.

### 2.2. PI3K/AKT Pathway

The PI3K/AKT signaling pathway is a central hub for mechanotransduction and a critical regulator of primordial follicle dormancy and activation [21]. It integrates mechanical and biochemical signals from ECM and GCs to maintain the ovarian reserve and support follicular development. This pathway is initiated by the interaction between Kit ligand (KitL), secreted by GCs, and the c-Kit receptor on oocytes [21]. Upon ligand binding, receptor tyrosine kinases activate phosphoinositide 3-kinase (PI3K), which catalyzes the production of plasma membrane lipid phosphatidylinositol 3,4,5-triphosphate (PIP3). PIP3 serves as a docking site for phosphoinositide-dependent kinase 1 (PDK1), which subsequently phosphorylates and activates the serine/threonine kinase AKT [22].

Phosphorylated AKT exerts its effects through multiple downstream targets, including the transcription factor FOXO3 (Figure 1). In its unphosphorylated state, FOXO3 resides in the nucleus and maintains follicular dormancy by repressing genes involved in oocyte growth and follicular activation. The phosphorylation of FOXO3 by AKT results in its cytoplasmic translocation, silencing its dormancy-promoting functions and initiating follicular activation [23,24]. In addition to FOXO3, the PI3K/AKT pathway influences follicular development through mTOR, a key downstream effector that regulates oocyte growth. AKT phosphorylates and inhibits the TSC1/TSC2 complex, thereby activating mTORC1 [25]. This activation drives processes such as protein synthesis, cell cycle progression, and metabolism, all essential for follicular development. mTOR activity also promotes the production of oocyte-derived factors like BMP15 and GDF9, which stimulate GC proliferation and maintain oocyte–GC communication throughout folliculogenesis [26,27]. Furthermore, mTOR-mediated suppression of autophagy in GCs protects against follicular atresia, further emphasizing the pathway’s role in preserving follicular viability [26].

The dysregulation of PI3K/AKT signaling has profound implications for ovarian function. The loss of PTEN, a negative regulator of this pathway, causes excessive primordial follicle activation, leading to the premature depletion of the ovarian reserve through atresia [28]. Conversely, PDK1 deficiency, which impairs pathway activation, results in the direct loss of dormant follicles without activation [29]. These opposing outcomes highlight the necessity of tightly regulated PI3K/AKT signaling for preserving the ovarian reserve. Additionally, microRNAs such as miR-20a and miR-494, which downregulate PTEN, enhance PI3K/AKT activity in GCs and promote oocyte maturation and follicle growth [30].

The PI3K/AKT pathway also integrates additional signaling cascades that contribute to follicular development. For instance, AKT-mediated phosphorylation and inactivation of GSK-3β have been implicated in oocyte growth and early follicle development, although the specific downstream targets of GSK-3β in this context remain to be fully elucidated [31]. Moreover, crosstalk between the PI3K/AKT and Hippo signaling pathways plays a significant role in regulating follicular activation, as these pathways collectively modulate the mechanical and biochemical cues within the ovarian microenvironment.

### 2.3. Hippo Pathway

**Figure 2:**
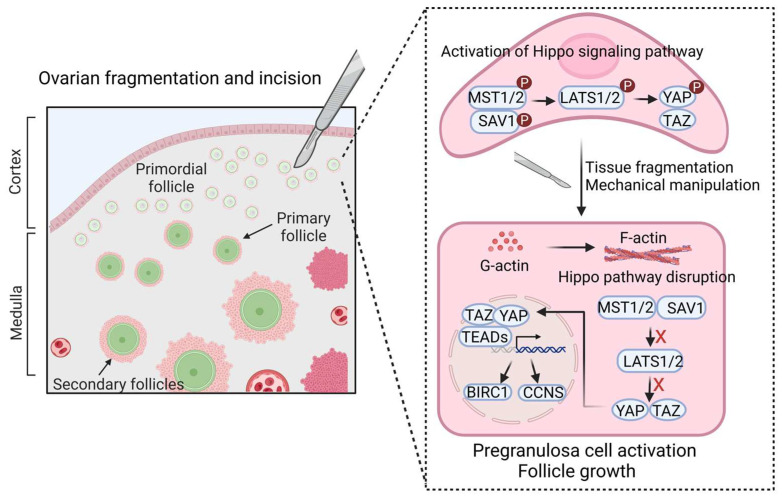
Tissue fragmentation and mechanical manipulation disrupt the ovarian Hippo signaling pathway, promoting follicle growth. In dormant primordial follicles, Hippo signaling maintains follicular quiescence by activating the MST1/2-SAV1 and LATS1/2 kinases, which phosphorylate YAP/TAZ in pre-granulosa cells (pre-GCs), leading to their cytoplasmic retention or degradation. This prevents YAP/TAZ from interacting with TEAD transcription factors, thereby suppressing growth-promoting gene expression. Ovarian fragmentation disrupts Hippo signaling by inducing actin polymerization, allowing YAP/TAZ to translocate into the nucleus. Once inside, YAP/TAZ proteins bind to TEADs to upregulate CCN growth factors and BIRC apoptosis inhibitors, stimulating granulosa cell proliferation, enhancing follicular survival, and promoting follicle activation. Created with BioRender.com.

The Hippo signaling pathway is a highly conserved mechanosensitive cascade that plays a pivotal role in regulating primordial follicle dormancy and activation [32]. In the ovary, active Hippo signaling helps maintain primordial follicle dormancy by phosphorylating and inactivating YAP and TAZ, thereby retaining them in the cytoplasm and preventing their nuclear localization. This process suppresses the expression of downstream growth-promoting genes such as CCN2 and BIRC, which are essential for cell proliferation and survival [33]. Mechanical disruptions, such as ovarian fragmentation or ECM remodeling, block Hippo signaling by promoting actin polymerization, reducing phosphorylated YAP levels, and facilitating the nuclear translocation of YAP/TAZ (Figure 2) [34,35]. Once localized in the nucleus, YAP/TAZ proteins interact with TEAD transcription factors to upregulate genes that promote granulosa cell proliferation, oocyte growth, and follicle activation [36,37]. These mechanotransduction events underscore the critical role of Hippo signaling in the regulation of ovarian reserve and the initiation of folliculogenesis.

Experimental studies have highlighted the therapeutic potential of manipulating Hippo signaling to activate dormant primordial follicles. Animal studies show that ovarian fragmentation induces actin polymerization, decreases p-YAP levels, and enhances YAP/TAZ nuclear localization, leading to increased expression of growth factors such as CTGF and apoptosis inhibitors like BIRC [32]. In mouse models, actin polymerization agents, such as sphingosine-1-phosphate (S1P), have been shown to promote Hippo pathway disruption, resulting in follicular activation and growth [32]. Additionally, combined approaches using ovarian fragmentation and autologous transplantation have successfully reactivated primordial follicles, improved ovarian function, and restored fertility [38].

### 2.4. Interplay Between PI3K/AKT and Hippo Pathways

The PI3K/AKT and Hippo signaling pathways exhibit significant crosstalk, forming a tightly regulated network that governs the balance between follicular dormancy and activation [39,40]. Both pathways integrate mechanical and biochemical cues from the ovarian microenvironment to ensure proper follicular dynamics, including the preservation of the primordial follicle pool and the activation of selected follicles. Studies have demonstrated that PI3K/AKT signaling modulates Hippo pathway activity by influencing YAP/TAZ dynamics. Specifically, the inhibition of AKT increases the phosphorylated-to-total YAP ratio, preventing YAP nuclear translocation and suppressing follicular activation [41]. This illustrates how AKT activity indirectly regulates YAP/TAZ transcriptional output, linking PI3K/AKT signaling to mechanotransduction processes mediated by the Hippo pathway. Conversely, the disruption of Hippo signaling, such as through ECM remodeling or ovarian tissue fragmentation, can enhance AKT-mediated pathways, promoting follicular activation (Figure 2) [42,43]. This crosstalk becomes particularly relevant in aging ovaries, where mechanical changes in the ECM disrupt the balance between these pathways [43].

The interplay between these pathways also coordinates GC and oocyte interactions during follicular development. AKT activation in GCs facilitates the production of growth factors such as BMP15 and GDF9, which are critical for sustaining oocyte–GC communication [18,19]. Simultaneously, YAP/TAZ signaling modulates GC proliferation and differentiation, ensuring proper follicular growth [36,37]. This coordination enables a seamless transition from dormancy to activation while promoting follicle survival.

Recent therapeutic approaches targeting this crosstalk underscore its clinical significance. For example, in women with premature ovarian insufficiency (POI), combined interventions involving AKT activators and Hippo signaling modulators have successfully induced follicular activation, leading to oocyte maturation and live births. These findings highlight the translational potential of leveraging the PI3K/AKT–Hippo pathway interplay to restore ovarian function and improve fertility outcomes.

## 3. Mechanobiology in Antral Formation and Ovulation

The antral follicle stage marks a pivotal transition from a secondary follicle to a large pre-ovulatory follicle, characterized by the accumulation of follicular fluid within the antrum [44]. This stage involves a dramatic increase in follicular volume, while the oocyte remains arrested in the prophase I stage of meiosis. Despite this meiotic arrest, the oocyte undergoes significant cytoplasmic growth, accumulating reserves of mRNA, proteins, and mitochondria necessary for resuming meiosis and supporting early embryonic development [45,46]. As the antrum forms, the follicle undergoes structural expansion and spatial repositioning within the ovary. This process is driven by a combination of ECM remodeling, osmotic gradients, and endocrine signaling pathways [47,48,49]. From a mechanobiological perspective, the dynamic remodeling of ECM components, along with their changing mechanical properties, provides structural integrity and signaling cues. These factors collectively support antrum formation and prepare the follicle for the mechanical demands of ovulation.

### 3.1. ECM Remodeling and Signaling Pathways in Antral Follicle Formation

ECM remodeling plays a dynamic regulatory role in follicular development, influencing the follicular structure, mechanical properties, and cellular differentiation. The basement membrane, primarily composed of collagens and laminins, undergoes continuous ompositional and structural modifications to accommodate the expanding follicle volume [50,51]. MMPs and a disintegrin and metalloproteinase with thrombospondin motifs (ADAMTS) proteases degrade and reorganize ECM components, such as collagens and proteoglycans, thereby modulating ECM mechanical properties and GC differentiation [52]. Specifically, ADAMTS proteases degrade basement membrane proteoglycans, such as decorin and versican, thereby regulating ECM stiffness and influencing granulosa-theca cell signaling [53]. Additionally, the ECM undergoes molecular modifications during follicular maturation. Laminin isoform transitions, including increased deposition of α2 and α4 chains, alter basement membrane permeability and elasticity, thereby modulating cell adhesion and signaling transduction [50,54]. The ECM modifications facilitate the stratification of GCs into mural GCs and cumulus cells, each playing distinct roles in follicular function and oocyte interactions. Collectively, ECM remodeling processes enable follicular expansion while maintaining structural integrity, supporting antrum formation, oocyte development, and mechanical preparation for ovulation [55].

Furthermore, multiple molecular mechanisms and signaling pathways coordinate antral follicle formation and expansion, contributing to follicular fluid accumulation and structural maturation. FSH stimulates the mTOR-CNP signaling pathway, upregulating aquaporins (AQP1, AQP2, and AQP5) in GCs to enhance transmembrane water transport, thereby facilitating antral cavity expansion [56]. Additionally, vascular endothelial growth factor increases capillary permeability, promoting the transfer of plasma solutes, hormones, and growth factors into the follicular fluid, providing essential nutrients and signaling molecules for oocyte development [3,57]. The PI3K/Akt pathway regulates GC proliferation and inhibits apoptosis, thereby promoting normal follicular growth and differentiation [58]. GCs secrete glycosaminoglycans and proteoglycans, generating osmotic pressure that supports antral cavity expansion [59].

As the antrum forms, multiple small fluid-filled cavities initially emerge and gradually merge into a single, continuous antral space. Throughout this process, intrafollicular pressure typically ranges between 15 and 20 mmHg but can transiently rise beyond 20 mmHg, reaching approximately 25 mmHg during rapid follicular expansion [60,61,62]. This mechanical environment influences GC morphology and function, reinforcing the structural integrity of the follicle. Simultaneously, GCs undergo spatial reorganization, with the innermost layer forming the cumulus oophorus, which surrounds and supports the oocyte. The integration of biochemical signaling, osmotic pressure, and mechanical forces collectively establishes the optimal environment for antral follicle maturation and subsequent ovulatory competence.

### 3.2. Mechanobiology of Ovulation

Ovulation is a mechanobiologically complex process requiring the integration of endocrine signals with localized mechanical forces to facilitate follicular rupture and oocyte release. The surge in luteinizing hormone (LH) initiates a cascade of cellular events that weaken the follicular apex, ultimately enabling follicular wall breakdown. At the molecular level, ECM degradation at the site of rupture is mediated by MMPs and serine proteases, which enzymatically degrade type IV collagen and fibrillar ECM components, reducing the tensile strength of the follicular wall [63,64,65]. Simultaneously, cumulus cell expansion, driven by hyaluronan synthesis and deposition, alters the extracellular microenvironment and facilitates oocyte detachment from the follicular wall [66].

The cytoskeletal dynamics of GCs play a crucial role in ovulation by modulating contractility and structural integrity. The RhoA/ROCK signaling pathway regulates actin remodeling within GCs, promoting contractility and mechanical stress at the follicular apex [67]. This localized contractile activity contributes to follicular wall thinning, enabling the extrusion of the cumulus–oocyte complex. In parallel, prostaglandin E2 (PGE2) and F2α (PGF2α) mediate follicular smooth muscle-like contractions, further facilitating rupture [66].

## 4. Mechanobiology in Ovarian Aging

Ovarian aging is characterized by a progressive decline in both the quantity and quality of ovarian follicles, leading to diminished ovarian reserve and disrupted meiotic maturation and fertilization, which are critical for successful reproduction [68,69,70,71,72,73]. While intrinsic cellular factors such as oxidative stress, mitochondrial dysfunction, and DNA damage contribute significantly to follicle and oocyte depletion [12,74,75,76], the role of the ovarian mechanical microenvironment is increasingly recognized as a critical driver of aging-related changes [4,7]. These mechanobiological changes during ovarian aging disrupt the physical and biochemical cues essential for maintaining the follicle reserve and supporting folliculogenesis, ultimately impairing oocyte quality and reproductive potential.

### 4.1. Altered ECM in the Aging Ovary

The ECM undergoes significant compositional and structural changes during ovarian aging [7,77]. Aging ovaries exhibit increased deposition of structural proteins, including collagen (COL1A1, COL3A1, and COL4A2), laminins (LAMA3 and LAMB1), and proteoglycans, along with a decline in elastin and fibronectin levels [7,78]. These alterations lead to increased tissue stiffness, which disrupts the delicate mechanical cues necessary to balance follicular dormancy and activation. Biomechanical studies suggest that the stiffness of the mouse ovarian cortex is approximately 2 kPa, while in aged ovaries, cortical stiffness increases to a range of 2–10 kPa, reflecting these structural changes and their impact on ovarian mechanics [7]. ECM remodeling enzymes, such as MMPs, play a critical role in maintaining ECM homeostasis. However, during aging, dysregulated MMP activity results in excessive ECM accumulation and stiffening, further contributing to ovarian dysfunction [78]. Additionally, glycosaminoglycans (GAGs) like hyaluronan (HA), which are vital for ECM hydration and elasticity, decline with age [7]. The concurrent generation of fragmented HA triggers inflammatory responses in ovarian stromal cells, destabilizing the follicular microenvironment and compromising the structural and functional integrity of the ovarian stroma [79].

The stiffened ECM imposes excessive mechanical stress on follicles, undermining their structural integrity and accelerating follicular atresia [80]. Age-related changes in ECM components, such as microfibril interface-located protein 1 (EMILIN-1), fibrillin-1, and other GAGs, weaken follicle–ECM interactions [11]. Furthermore, the accumulation of advanced glycation end-products (AGEs) exacerbates ECM rigidity by cross-linking ECM proteins, reducing their flexibility and regenerative capacity [81]. This dysregulated ECM remodeling impairs granulosa cell–oocyte communication, disrupts normal follicular progression, and destabilizes follicle architecture [52].

### 4.2. Follicular Atresia and Ovarian Reserve Decline During Aging

Follicular atresia is the primary contributor to ovarian reserve depletion during aging, driven by both intrinsic cellular dysfunctions and extrinsic mechanical disruptions. Intrinsically, oocytes and GCs become increasingly prone to degeneration due to elevated oxidative stress, mitochondrial deterioration, and dysregulated signaling pathways [82,83]. These cellular impairments weaken their ability to maintain follicular integrity and resilience, accelerating the attrition of ovarian follicles. Extrinsically, the accumulation of fibrotic ECM and stiffened ovarian stroma exacerbates follicular attrition by imposing mechanical stress on the ovarian microenvironment [11,84]. The dense and rigid ECM forms a physical barrier that limits the diffusion of essential nutrients, oxygen, growth factors, and hormones to the follicles. This disruption undermines GC–oocyte communication and impairs mechanosensitive signaling pathways critical for follicular survival. As a result, the hostile mechanical environment destabilizes the follicular architecture, hinders follicle progression through developmental stages, and accelerates atresia [85]. Furthermore, the excessive accumulation of fibrotic ECM not only increases tissue stiffness but also disrupts ECM remodeling, a dynamic process vital for healthy folliculogenesis. Dysregulated ECM turnover impairs the balance between structural support and tissue flexibility, further compromising the ovarian microenvironment. Preclinical studies have shown that anti-fibrotic therapies, such as pirfenidone and BGP-15, can effectively reduce fibrosis, improve mitochondrial function, and restore folliculogenesis in aged mice [86]. These findings underscore the pivotal role of ECM dynamics in determining follicular fate during aging and highlight the potential of targeting mechanobiological pathways as a therapeutic strategy to mitigate ovarian aging and preserve reproductive health.

### 4.3. Mechanobiology-Based Interventions to Counteract Follicle Loss

Recent advancements in mechanobiology have led to innovative strategies to mitigate follicular atresia and support follicular growth in aging ovaries [78,87,88]. These interventions leverage insights into mechanical forces, ECM dynamics, and mechanosensitive signaling pathways to create a more supportive ovarian microenvironment for folliculogenesis. By addressing both structural deficiencies in the ECM and dysregulated signaling pathways, these approaches aim to restore follicular function and improve reproductive outcomes.

One of the most promising interventions is in vitro activation (IVA), a technique that combines the mechanical fragmentation of ovarian cortical tissue with the pharmacological activation of the PI3K/AKT signaling pathway [39,40]. Mechanical fragmentation disrupts the Hippo signaling pathway by promoting actin polymerization and the nuclear translocation of YAP, a key activator of follicular growth [89]. Simultaneously, the pharmacological activation of PI3K/AKT enhances cellular growth and survival, effectively reactivating dormant follicles [90,91,92]. Clinical studies have demonstrated IVA’s success in improving live birth rates among women with POI and diminished ovarian reserve (DOR), positioning it as a transformative therapeutic strategy [90,93]. A simplified, drug-free version of IVA relies solely on mechanical fragmentation, eliminating the use of pharmacological agents. While its efficacy and safety require further validation, this approach has shown promise in increasing antral follicle counts and achieving live births, offering a less complex and accessible alternative for clinical use.

Whole ovary laparoscopic incision (WOLI), another innovative intervention, is a minimally invasive surgical technique designed to alleviate ovarian fibrosis and ECM stiffness, two hallmarks of ovarian aging and diminished function [94]. This procedure involves creating parallel streak incisions on the ovarian cortex, thereby reducing mechanical stress, improving vascularization, and enhancing nutrient and oxygen diffusion throughout the ovarian microenvironment. Clinical evidence suggests that WOLI stimulates the growth of small antral follicles, improves hormonal responses, and rejuvenates ovarian function in women with extremely poor ovarian response (EPOR) [94]. By directly targeting mechanical constraints within the ECM, WOLI restores a more dynamic and permissive environment for follicular development.

The effectiveness of both IVA and WOLI lies in their ability to modulate ovarian ECM mechanics and reestablish critical signaling pathways. These mechanical interventions alleviate the stiffness of the fibrotic ECM, reactivating mechanosensitive pathways such as Hippo and PI3K/AKT, which regulate follicular dormancy and growth. Furthermore, by reducing hypoxia and nutrient diffusion barriers caused by the dense ECM, these approaches facilitate the delivery of essential resources to growing follicles [95]. Techniques like WOLI also improve vascular supply and trigger controlled inflammatory responses that promote ECM remodeling, creating a rejuvenated ovarian microenvironment conducive to folliculogenesis.

## 5. Emerging 3D Culture Models

**Figure 3:**
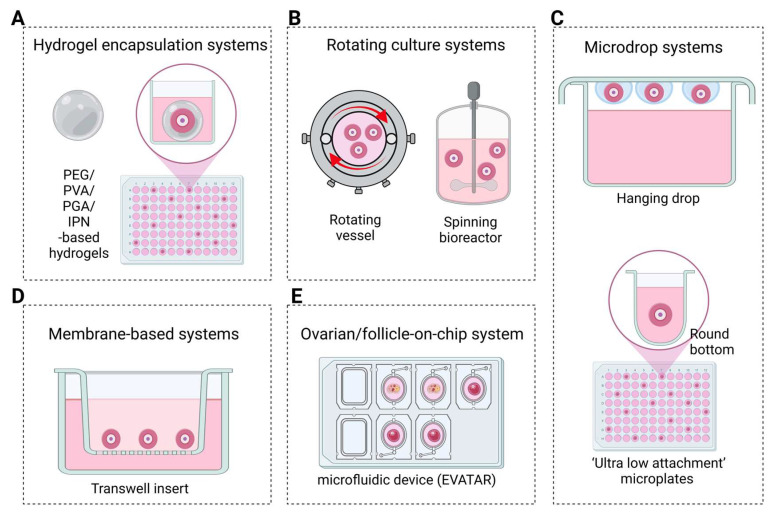
Schematic representation of various 3D culture techniques for ovarian follicle culture. (**A**) Hydrogel encapsulation systems: natural or synthetic hydrogels mimic the ovarian extracellular matrix (ECM), providing structural support and biochemical cues essential for follicular growth. (**B**) Rotating culture systems: spinner flasks and rotating wall vessels enhance nutrient diffusion, oxygen exchange, and shear stress exposure, preventing follicle aggregation and promoting optimal development. (**C**) Hanging drop and ultra-low attachment plates: hanging drop cultures suspend follicles in droplets to maintain their 3D structure, while ultra-low attachment plates prevent adhesion, preserving the follicular architecture. (**D**) Transwell insert culture: follicles are cultured on a microporous membrane, enabling bidirectional nutrient exchange and mimicking in vivo metabolic interactions while supporting follicular integrity. (**E**) EVATAR system: a microfluidic model of the human reproductive tract that connects ovarian, fallopian, and uterine tissues, allowing for the real-time study of endocrine, immune, and metabolic influences on follicular development. Created with BioRender.com.

Advanced 3D ex vivo culture models, including the follicle-on-a-chip and ovary-on-a-chip platforms, have become critical tools for optimizing mechanopharmacological interventions (Figure 3). These innovative systems replicate the in vivo mechanical and biochemical properties of the ovarian ECM, allowing researchers to evaluate how specific compounds influence ECM flexibility, granulosa cell function, and overall follicular health [96,97]. Diverse 3D culture methods, such as hydrogel encapsulation, membrane-based setups, rotating systems, and microdrop systems, offer unique advantages for studying follicle growth, steroidogenesis, and pharmacological responses aimed at enhancing oocyte quality and extending reproductive longevity.

### 5.1. Hydrogel Encapsulation Systems

Hydrogel encapsulation is among the most widely used 3D culture methods, offering both natural and synthetic hydrogels designed to mimic the ovarian ECM (Figure 3). These hydrophilic polymers absorb large amounts of water, providing a supportive matrix that ensures oxygen and nutrient transport while preserving the follicular structure [98]. Synthetic hydrogels, such as polyethylene glycol (PEG), polyvinyl alcohol (PVA), and polyglycolic acid (PGA), provide high mechanical strength and precise control over physical properties [99,100,101]. However, their limited bioactivity and biodegradability may restrict the dynamic cell interactions necessary for follicular expansion and function.

Natural hydrogels, including alginate, collagen, fibrin, and hyaluronic acid (HA), possess inherent bioactive properties that support cell viability and function. Alginate hydrogels, derived from brown algae, have been widely utilized for ovarian follicle encapsulation, providing a stable and reproducible environment that preserves follicular shape and facilitates granulosa cell–oocyte interactions [4,102]. However, alginate’s non-degradable nature limits follicular expansion, prompting the development of advanced materials like fibrin–alginate interpenetrating (FA-IPN) [103] and alginate–reconstituted basement membrane (Alg-rBM IPN) networks [4]. FA-IPN hydrogels allow for partial degradation during follicle growth, improving oocyte maturation and viability. Similarly, Alg-rBM IPN networks combine alginate with basement membrane components to enhance the bioactivity and mechanical properties of the hydrogel, providing a more supportive environment for follicular development [4].

Advancements in hydrogel technology include PEG-based hydrogels, which provide tunable mechanical properties to accommodate follicle volume increases while preserving the 3D architecture [104]. These hydrogels improve follicle survival and oocyte development, supporting the maturation of oocytes with enhanced developmental competence. Similarly, HA-based hydrogels provide superior biocompatibility and bioactivity. HA promotes structure and homeostasis. When incorporated into hydrogel systems, HA supports follicular growth, estradiol production, and oocyte maturation. Its plasticity and viscosity make HA hydrogels particularly suited for long-term follicle culture and drug testing applications [105].

### 5.2. Rotating Culture Systems

Rotating follicle culture systems employ gentle orbital movement to suspend follicles in a liquid medium, preventing adhesion to the culture vessel surface. Initially explored in test tubes, this technique uses controlled rotation to sustain follicle suspension and enhance nutrient exchange. Rowghani et al. demonstrated that rotating wall vessels—cylindrical chambers with axial rotation—preserve the 3D architecture critical for maintaining follicular shape and cell–cell interactions [106]. However, high rotation rates can damage follicle integrity, highlighting the need for the careful optimization of rotation speed to support follicle health. These systems provide a stable 3D environment conducive to follicle development, offering a promising platform for drug screening. By preventing substrate attachment, rotating systems enable follicles to retain their natural structure and interactions, which are essential for evaluating the effects of anti-aging compounds and other therapeutic agents on follicle growth and oocyte quality [106,107,108].

### 5.3. Microdrop and Membrane-Based Systems

Microdrop-based culture systems provide an effective method for in vitro follicle culture. These systems use small droplets of a culture medium containing individual follicles placed on non-adhesive, hydrogel-coated plates to prevent cell attachment. This setup preserves the follicular architecture, supports steroidogenic activity, and enhances blastocyst yield, demonstrating its effectiveness in promoting follicular maturation [109]. Modified approaches, such as the inverted microdrop technique, position droplets on inverted plates, allowing follicles to sink and achieve uniform nutrient and oxygen exposure, resulting in faster growth and increased estradiol production [110,111].

Another approach within the floating system category utilizes microporous membranes to create a permeable interface, exposing the entire follicle surface to the culture medium. This setup facilitates metabolic exchanges akin to in vivo conditions, thereby enhancing follicle growth and maturation [111,112,113,114]. For example, O’Brien et al. demonstrated the effectiveness of microporous membranes in culturing mouse ovarian follicles, which led to the production of viable offspring [114]. These findings underscore the potential of membrane-based systems in supporting complete folliculogenesis.

### 5.4. Ovarian Follicle-on-Chip System

Ovarian follicle-on-chip systems represent a significant advancement in reproductive biology, combining microfluidic technology with 3D culture environments to closely mimic the ovarian microenvironment [115,116]. These platforms offer precise control over mechanical and biochemical conditions, facilitating detailed studies of follicular development and function. One notable approach involves using a microfluidic flow-focusing device to encapsulate early secondary preantral follicles within core–shell microcapsules, creating biomimetic ovarian microtissue [117]. This method has been shown to support follicular growth and maturation effectively. Additionally, studies have demonstrated that microfluidic platforms can replicate traditional dish-based follicle cultures, indicating their potential as reliable alternatives for in vitro studies [118].

Advancements in this field include the development of multi-unit microfluidic platforms capable of simulating the human reproductive tract and menstrual cycle. Xiao et al. designed a microfluidic system that supports murine ovarian follicles in producing the human 28-day menstrual cycle hormone profile. This hormone profile regulates the dynamics of the human female reproductive tract and peripheral tissues, providing a platform for studying endocrine, immune, and metabolic factors influencing follicular development across single-, dual-, and multiple-unit systems [119]. Additionally, Laronda et al. integrated 3D printing technology to create bioprosthetic ovaries using microporous scaffolds. These scaffolds successfully restored ovarian function in sterilized mice, demonstrating their potential for fertility preservation and restoration [120]. By supporting follicular development and hormone production, bioprosthetic ovaries highlight the transformative potential of such technologies in addressing reproductive challenges.

### 5.5. Applications in Mechanopharmacological and Reproductive Medicine

The ability of 3D culture systems to replicate in vivo mechanical and biochemical properties is critical for advancing mechanopharmacological research. PEG and HA hydrogels, with their ECM-mimetic properties, enable precise investigations into how ECM stiffness affects drug efficacy and cellular behavior. Advanced models like follicle-on-a-chip, incorporating microfluidic technology, facilitate real-time monitoring of follicle development, hormone cycles, and therapeutic impacts, making them robust tools for personalized medicine. These systems are particularly suited for testing anti-fibrotic drugs like pirfenidone and mitochondrial-targeted agents such as BGP-15 under physiologically relevant conditions.

**Figure 4:**
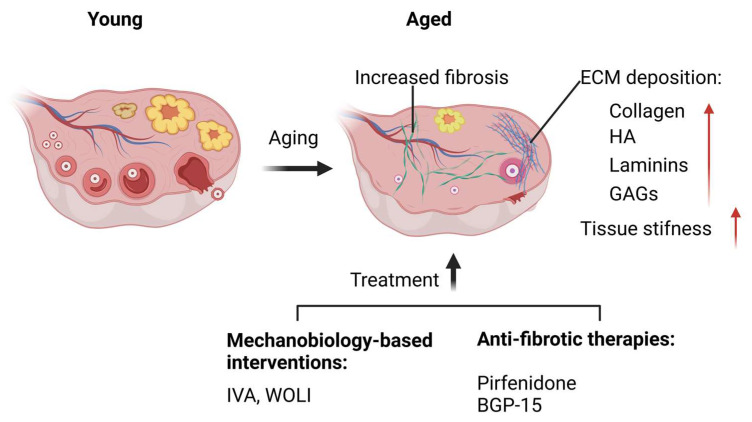
Schematic representation of ovarian aging and potential therapeutic interventions. Aging leads to increased fibrosis, excessive extracellular matrix (ECM) deposition, and heightened tissue stiffness, primarily due to the accumulation of collagen, hyaluronic acid (HA), laminins, and glycosaminoglycans (GAGs). These structural alterations contribute to follicular depletion and ovarian dysfunction. Mechanobiology-based interventions such as in vitro activation (IVA) and whole ovary laparoscopic incision (WOLI) aim to counteract these aging effects by modulating mechanical cues and restoring follicular growth. Additionally, anti-fibrotic therapies, including Pirfenidone and BGP-15, target ECM remodeling to alleviate fibrosis and improve ovarian microenvironmental conditions, thereby supporting folliculogenesis and preserving ovarian function. Created with BioRender.com.

Recent studies have demonstrated that pirfenidone, an FDA-approved antifibrotic agent, effectively reduces ovarian fibrosis and mechanical stress in aged ovaries, thereby restoring follicular survival and oocyte quality [86,121]. Mechanistically, pirfenidone attenuates TGF-β-induced fibrotic remodeling and ECM stiffness, rescuing ovarian microenvironment integrity. Similarly, BGP-15, a mitochondrial-protective agent, has been shown to enhance mitochondrial function and reduce oxidative stress in ovarian cells, improving follicular viability and oocyte competence [86,122]. By integrating such compounds into advanced 3D culture models, researchers can refine therapeutic strategies aimed at modulating ovarian mechanobiology to preserve reproductive function. These approaches hold promise for mitigating age-related infertility and optimizing assisted reproductive technologies (ARTs) (Figure 4).

## 6. Conclusions and Future Directions

Mechanobiology has revolutionized our understanding of ovarian follicle dynamics, highlighting the critical role of mechanical forces, ECM properties, and their interplay with intracellular signaling pathways in regulating follicular dormancy, activation, and development. Mechanical cues such as ECM stiffness, compressive forces, and shear stress are integral for maintaining the ovarian reserve, supporting folliculogenesis, and ensuring reproductive longevity. Disruptions in these mechanical environments, whether due to aging, fibrosis, or pathological conditions, compromise follicular health and lead to premature activation, atresia, and ovarian reserve depletion. Emerging therapeutic approaches, such as IVA and WOLI, combined with advanced 3D culture systems, have demonstrated great promise in restoring follicular function and improving fertility outcomes by targeting the mechanobiological microenvironment (Figure 4).

Looking ahead, integrating multi-omics approaches with advanced imaging and biomechanical analyses will provide deeper insights into mechanosensitive pathways and help identify novel biomarkers and therapeutic targets for ovarian health. Personalized mechanopharmacology, leveraging patient-specific 3D culture systems and microfluidic platforms, holds the potential to tailor treatments for conditions like premature ovarian insufficiency and diminished ovarian reserve. Additionally, exploring aging-induced mechanobiological changes in ECM composition and cellular signaling could inform interventions aimed at delaying ovarian aging and extending reproductive longevity. Innovations such as follicle-on-chip systems and bioprosthetic ovaries, supported by 3D printing and bioengineering technologies, offer transformative possibilities for fertility preservation and mechanobiology-driven drug discovery. By bridging basic research with clinical application, mechanobiology is poised to redefine reproductive medicine and provide solutions for improving fertility and ovarian health.

## Data Availability

Not applicable.
